# Enhanced sonocatalytic degradation of diethylstilbestrol using multi-walled carbon nanotubes under ultrasonic irradiation^☆^

**DOI:** 10.1016/j.ultsonch.2026.108084

**Published:** 2026-10-01

**Authors:** Manman Sun, Yihan Sun, Jingzhen Wang, Shengkun Tang, Hongyu Chen

**Affiliations:** a School of Physics and Telecommunications Engineering, Zhoukou Normal University, Zhoukou 466001, China; b College of Chemistry and Chemical Engineering, Zhoukou Normal University, Zhoukou 466001, China

**Keywords:** Diethylstilbestrol (DES), Multi-walled carbon nanotubes (MWCNTs), Sonocatalysis, Hydroxyl radicals, Advanced oxidation process, Water treatment

## Abstract

The continuous release of endocrine-disrupting compounds (EDCs) into Aquatic habitats have emerged as a considerable environmental issue due to their persistence, propensity for bioaccumulation, and detrimental biological consequences, even at low concentrations. Among these contaminants, diethylstilbestrol (DES), a synthetic estrogen, has attracted considerable attention because of its strong endocrine-disrupting activity and resistance to conventional wastewater treatment technologies. This study exploited multi-walled carbon nanotubes (MWCNTs) as sonocatalysts to further enhance the ultrasonic degradation of DES in educational and aquatic environments. The physiochemical properties of MWCNTs were determined using BET surface area measurements, Raman spectroscopy, and determination of the point-of-zero-charge (pHPZC). BET analyses proved the porous structure of the catalyst with pore size distribution ranging mainly from 35-45 nm. From Raman spectroscopy, well defined D, G, and 2D bands at 1338, 1584 and 2692 cm^−1^ respectively were observed with ID/IG ratio 0.82 suggesting creation of active defect sites for catalyst activity. Studies were carried out with respect to varying operating parameters like liquid height, stirring speed, pH, temperature, initial DES concentration. It was found that the proposed system (ultrasonic-MWCNTs hybrid system) showed significantly higher efficiency than alone adsorption or only sonication. Optimal parameters for degradation were found to be 5 mm liquid height and 300 rpm stirring speed. From radical scavenging experiment, the reactive radical for the degradation of DES was found to be hydroxyl radicals (OH). It was also found that MWCNTs was very stable up to 5 cycles of application. The research confirmed that MWCNTs-assisted sonocatalysis is an effective and green process for removal of endocrine-disrupting pollutant from water systems.

## 1 Introduction

Over the recent decade, widespread presence of endocrine-disrupting chemicals (EDCs) in aquatic systems became a significant worldwide environmental and health concern. Low-concentration level of EDCs may cause adverse effects on reproductive, developmental, neural and immunologic system of human and wild life via disrupting hormonal signal pathways. The constant release of drugs, industrial compounds, personal hygiene products and agricultural poison into the water body has resulted in overwhelming the aquatic ecosystem with contamination load. EDCs have been consistently detected in river, lakes, groundwater, wastewater treatment plant effluents, drinking water due to its persistency, bioaccumulation, and Biological Activity [1], [2], [3].

Among various endocrine-disrupting compounds, one of the most potent synthetic estrogens discovered is Diethylstilbestrol (DES). DES is a synthetic, non-steroidal estrogen compound which was extensively prescribed for pregnant women and widely used in veterinary medicine for promote animal growth. Even though it is banned in many countries due to its carcinogenic and reproductive hazard properties, it still can be found in many environmental matrices owing to improper disposal, industrial pharmaceutical wastewater discharge and past contamination. Various toxicology studies have correlated the effects of DES exposure with the reproductive abnormalities, developmental malformation, infertility, endocrine dysfunction, and higher risk to breast, vaginal, and prostate cancers [6], [9], [14]. The methods of treatment of industrial waste have included biological degradation, coagulation-flocculation, membrane filtration, adsorption and biological oxidation. All of them are primarily used to remove organic pollutants but in effective removal of endocrine-disrupting chemicals, its efficiency is limited in terms of total removal. Hence there is an urgent need for developed sophisticated treatment methods that can convert DES to other less toxic compounds or achieves total mineralization [1], [2], [4]. Advanced oxidation processes (AOPs) have attracted researcher to employ the technology for the degradation of recalcitrant organic pollutants through formation of various active oxygen species. Hydroxyl radicals (OH), have highest oxidation potential (∼2.8 V) among the highly oxidative species and widely used in water treatment applications. Multiple advanced oxidation techniques, including photocatalysis, Fenton oxidation, ozonation, electrochemical oxidation, and sonochemical oxidation, have shown beneficial for wastewater treatment [4], [11], [14].

Sonocatalysis as a green advanced oxidation technology utilizes the acoustically-induced cavitation effect created by the ultrasonic wave which results in the formation and collapsing of the bubbles creating a rising temperature and pressure localised hotspots facilitating improvement of •OH, •H, H2O2, O2•−. Other process occurring in sonolysis, that is acoustic wave effect, which induces microjets and shock waves, enhances mass transfer and kinetics [5], [7], [10]. Although the efficiency of sonolysis for the destruction of pollutants was observed to be efficient but rapid recombination of radicals and poor radical production limited its effective removal. Catalysts can be incorporated into sonication processes to increase the catalytic processes known as sonocatalysis. Addition of catalyst can enhance cavitation intensity, radical production and pollutant-catalyst interaction. As a result, significant improvements in reducing organic contaminants will be obtained [5], [10], [13].

Carbon nanoparticles were considered attractive catalytic materials in sonocatalysis over many other materials based on their large surface area, excellent electric conductivity and chemical stability. Recently, multi-walled carbon nanotubes (MWCNTs) were of growing interest because of their novel tubular nanostructure, excellent mechanical properties, and abundant active adsorption sites [8], [12], [28]. The combination of multi-walled carbon nanotubes (MWCNTs) with an ultrasound system improved the degradation of pollutant through increasing the adsorption rate, owing to their large surface area and their function as active nucleation sites, promoting the cavitation induced bubble nucleation and collapse. Although the MWCNTs have been widely studied as adsorbents and carbon-based catalytic materials for removing organic contaminants because of their high specific surface area, tunable surface chemistry and structural stability, their application in ultrasound-assisted degradation of diethylstilbestrol (DES) remains relatively limited. The combination of MWCNTs and ultrasonic irradiation, in particular, offers a unique chance to combine the nanotube surface adsorption capacity and the formation of reactive species during the acoustic cavitation. MWCNTs also can be active interfacial sites for cavitation bubble nucleation and increase the proximity between adsorbed DES molecules and reactive oxygen species, which promotes the oxidative degradation. Therefore, the present study aims to develop a MWCNT-assisted sonocatalytic system for degradation of DES and to systematically assess the impact of key operational parameters, involvement of reactive species, catalyst reusability and performance in a realistic river-water matrix. This integrated approach overcomes the limited investigation of MWCNT-mediated sonocatalysis specifically for the removal of DES and provides mechanistic and operational insights relevant to the development of advanced treatment strategies for endocrine disrupting contaminants.

Physicochemical properties such as nitrogen adsorption–desorption, Raman spectra and pHPZC determination were reported to provide an insight of degradation mechanism and optimization of catalyst efficiency [12], [13], [28]. Studies focusing on the degradation of DES using MWCNTs assisted sonocatalysis are relatively limited, therefore development of an efficient MWCNT assisted sonocatalytic system for degradation of DES marks a promising step towards development of sustainable water treatment technology [10], [27], [28].

## 2 Materials and methods

### 2.1 Chemicals and Reagents

Diethylstilbestrol (DES, purity ≥ 99%), an endocrine disruptor, was purchased from SigmaAldrich (St. Louis, MO, USA). Multiwalled carbon nanotubes (MWCNTs) with outer diameter of 1020  nm, length of 1030  μm and purity of 95% were purchased from SigmaAldrich.Methanol (HPLC grade), acetonitrile (HPLC grade), benzoic acid, tertbutanol (tBuOH), hydrochloric acid (HCl), sodium hydroxide (NaOH), phosphoric acid, sodium phosphate monobasic and sodium phosphate dibasic were obtained from Merck (Darmstadt, Germany).5,5dimethyl1pyrrolineNoxide (DMPO), the spintrapping reagent for electron paramagnetic resonance (EPR) was from Tokyo Chemical Industry (Tokyo, Japan).Ultrapure deionized (DI) water with a resistivity of 18.2 MΩcm purchased from a MilliQ water purification system was used for all parts of the entire study.Stock solution of DES (1000  mg L^-1^) was prepared in methanol and stored in amber glass containers at 4oC. The stock solution was freshly diluted in deionized water immediately prior to each experiment to give a working solution.

### 2.2 Synthesis and functionalization of MWCNTs

Prior to sonocatalytic experiments, commercial MWCNTs were purified and functionalized to improve dispersion, increase active surface sites, and remove residual metal impurities. 1 g of MWCNTs was dispersed in 3:1 mixture solution of concentrated sulfuric acid (H2SO4) and nitric acid (HNO3). The suspension was sonicated for 2 h. The solution was then mechanically stirred at 70C for 6 h. The acid-treated MWCNTs were washed by the deionized water several times until the pH value of filtrates was neutral. The purified MWCNTs were obtained by vacuum filtration, and dried at 80C for 12 h in the hot air oven. The dry powders were finely ground in agate mortar by careful rubbing and stored in airtight containers for future use. The presence of oxygen-containing functional groups (e.g. hydroxyl group-(OH), carbonyl group-(C=O) and carboxyl group-(COOH)) on the nanotube surface resulted in better catalysts' dispersion and enhancement of their sonocatalytic activity due to acid treatment.

### 2.3 Experimental Procedure and Apparatus

Sonocatalytic degradation experiments were carried out in a 970 kHz high frequency ultrasonic reactor. Ultrasonic irradiation was done at the same frequency of 970 kHz. In the present study the ultrasonic power density (WL^−1^) was not evaluated individually so this is designated as one restriction in the scale-up and reproduction. The ultrasonic set-up consisted of a stainless-steel ultrasonic bath with a recirculating chiller with temperature control to keep a constant reaction temperature throughout the experiment. The 250 mL borosilicate glass reactor with 100 mL of DES solution was positioned in the middle of the ultrasonic bath. The required amount of MWCNT catalyst was added to the reactor and the suspension was magnetically stirred in the dark for 30 min for adsorption–desorption equilibrium to be reached prior to sonication treatment. A parametric study was performed to examine the effect of various operating conditions including initial DES concentration (2–30 M), catalyst dosage (50–500 mg/L-1), solution pH (4, 7 and 10), stirring speed (0–400 rpm) and temperature (25–45°C). The operating ranges studied were chosen to include conditions that are typically relevant to ultrasonic advanced oxidation and to permit systematic investigation of the effects of acoustic energy distribution, hydrodynamic mixing, catalyst surface interactions, and reaction temperature. The effect of ultrasonic attenuation and acoustic energy transfer in the reaction medium was studied by changing the liquid height over a wide range. To determine the balance between MWCNT dispersion, pollutant mass transfer and cavitation stability, the stirring speed was changed from static conditions to vigorous mixing. Solution pH of 4, 7 and 10 were chosen to simulate acidic, near neutral and alkaline conditions and to evaluate the effect of surface charge of the catalyst and interactions between the pollutants and catalyst. The temperature range of 25–45°C was chosen to study the effect of moderate temperature increase on reaction kinetics without excessive heating that can negatively affect cavitation. Samples were removed at predetermined intervals (0–60 min) during sonication and filtered with a 0.22 m membrane filter to separate the catalyst particles prior to their analysis.

Residual amount of DES was determined by using high-performance liquid chromatography (HPLC; Agilent 1260 Infinity, USA) with diode array detector using Zorbax Eclipse Plus C18 column (4.6 mm 150 mm, 5 m) and the mobile phase of acetonitrile and water was 70:30 v/v at a flow rate 1.0 ml min1. Injection of DES was 20 l and the measurement of wavelength was done at 240 nm. The efficiency (%) of DES degrading was calculated as follows:

Degradation efficiency (%) = (C0 − Ct)/C0 × 100.where C0 is the initial concentration of DES (μM), Ct is the concentration of DES at reaction time t (μM).

The degradation kinetics was studied by pseudo-first order kinetic model:

ln (Ct/C0) = -kobs t. kobs is the observed first order rate constant (min − 1), and t is the reaction time (min).

The synergistic index (SI) of ultrasound assisted MWCNT system was calculated as:

SI = k (US/MWCNT) / [k (US) + k (MWCNT)]where k is the pseudo-first-order degradation rate constant determined for each individual process.

### 2.4 Characterization of Multi-Walled carbon nanotubes (MWCNTs)

Some methods were carried out to describe the physical properties of MWCNTs. Field emission scanning electron microscopy (FESEM JEOL JSM-7600F, Japan) was employed to study the morphology of MWCNTs. The elemental composition of MWCNTs was tested by field emission scanning electron microscopy (FESEM) and Energy dispersive X-ray spectroscopy (EDS). The graphitic ordering and structural defects of MWCNTs were evaluated through determining the intensity ratio of the D band to the G band (ID/IG) by Raman spectroscopy (Horiba LabRAM HR Evolution, France) at the 532 nm laser. BET nitrogen adsorption–desorption method was applied to determine the specific surface area, pore volume and pore size distribution (Micromeritics ASAP 2460, USA). The elemental composition of MWCNTs was studied by Field Emission Scanning Electron Microscopy (FESEM) and Energy Dispersive X-ray Spectroscopy (EDX). X-ray diffraction (XRD, Rigaku Ultima IV, Japan) with Cu-K radiation (= 1.5406) in scanning range 10–80 (2) was used to analyse crystalline structure. DLS was used to determine the size distribution of MWCNT prepared. Before analysis, MWCNT samples were heated up in vacuum for 12 h to get rid of moisture or contaminants at 120°C. The pHpzc was determined using the pH drift method. Meanwhile, Benzoic acid was employed as a molecular probe for hydroxyl radical generation from p-hydroxybenzoic acid (p-HBA).

### 2.5 Experiments with river water

Water samples were utilized to check the efficiency of the sonocatalytic system. River water samples were promptly brought to lab and filtered with 0.22 m nylon membrane filter to remove impurities, suspended materials and the debris of water samples and then measured of physicochemical properties of river water, such as pH, conductivity, TDS, TOC and major inorganic species before sonocatalytic test were performed. Filtered river water stored in refrigerator under 4C was used within 48 h for treatment after which they were not used for further treatment. For sonocatalytic degradation tests, DES was added in the river water medium under different values in accordance with the lab scale results. The effect of coexisting species and natural organic matter on the MWCNT-assisted ultrasonic sonocatalytic system was evaluated by comparing removal efficiency of DES in river water and DI water. Performance under different treatment conditions, including removal efficiency of DES, mineralization rate and catalyst reuse under optimal treatment conditions, the robustness and feasibility of the sonocatalytic process in realistic environment water matrix, were performed to evaluation.

### 2.6 Statistical analysis

All experimental results are from triplet and mean + SD. Statistical significance was calculated using one-way ANOVA. P < 0.05 was designated as statistically significant. p < 0.001, p < 0.0001.

## 3 Results and Discussion

### 3.1 Characterization

The produced MWCNTs were characterized by several characterization techniques to evaluate their suitability as sonocatalysts for DES degradation. Their physicochemical properties were investigated **(**Fig. 1). The FESEM images showed highly entangled tubular morphology with interconnected nanotube networks. The nanotubes exhibit consistent diameter within the range of 10–20 nm (Fig. 1a) and thus it has a very high outer surface area, which allows effective adsorption of pollutants and generation of cavitation bubbles.

**Fig. 1:**
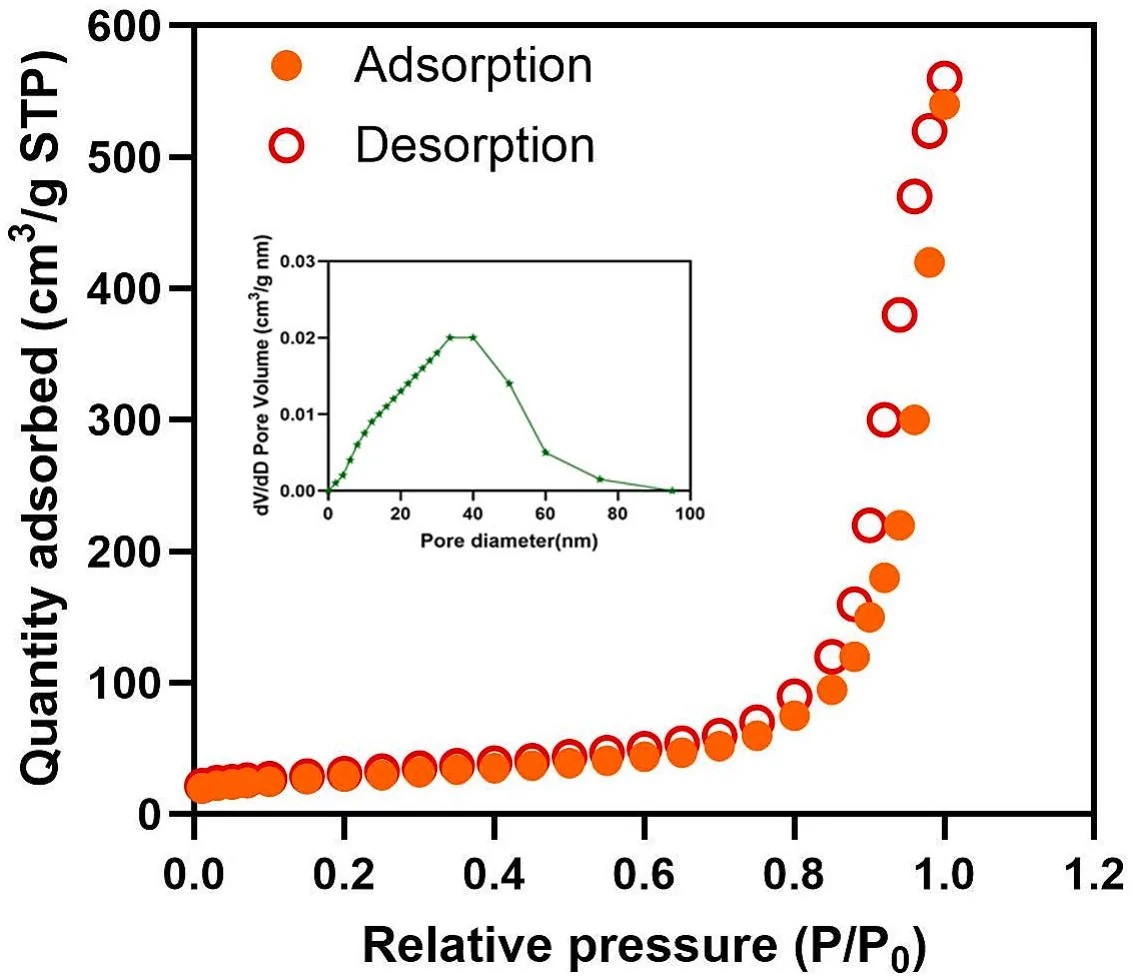

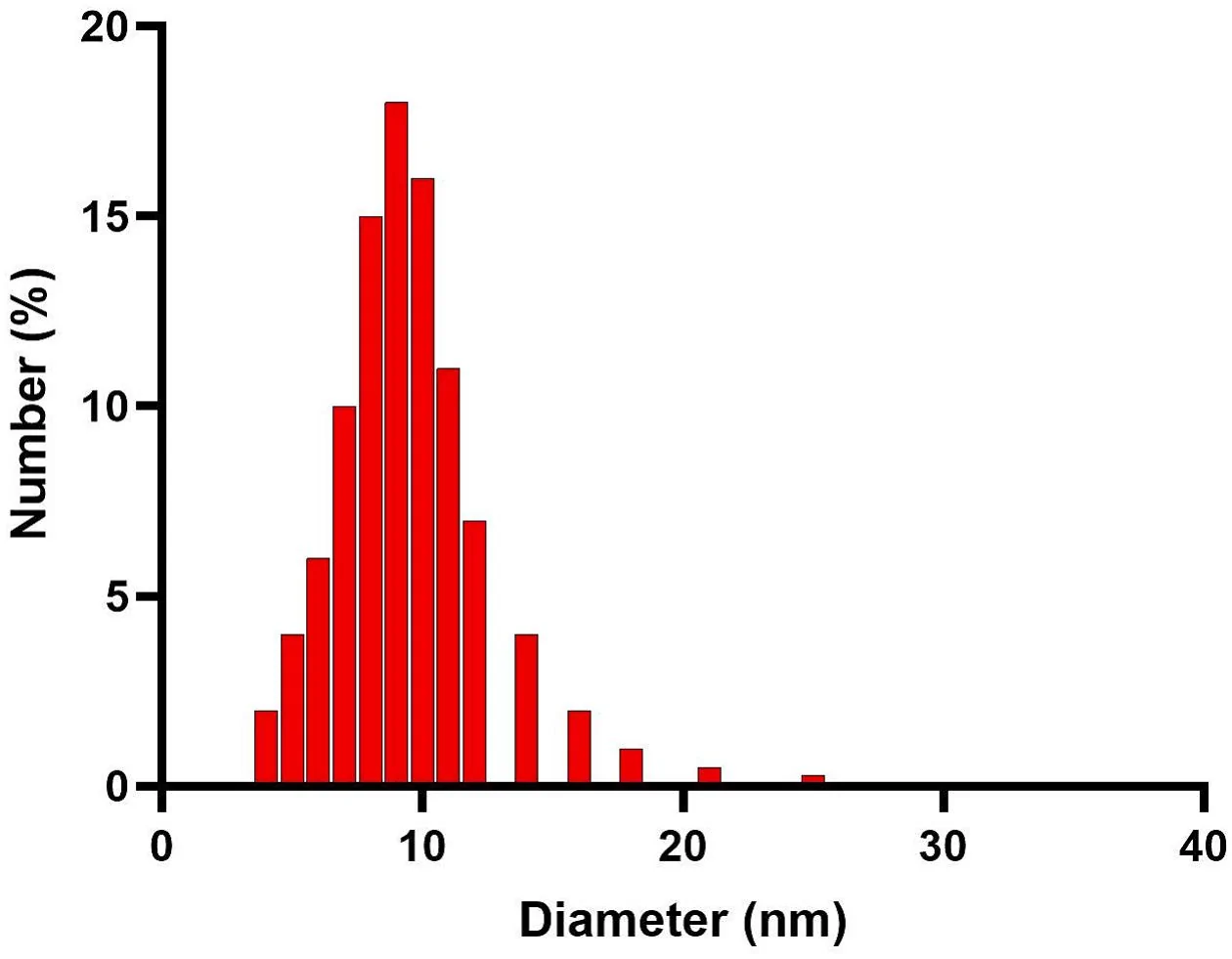

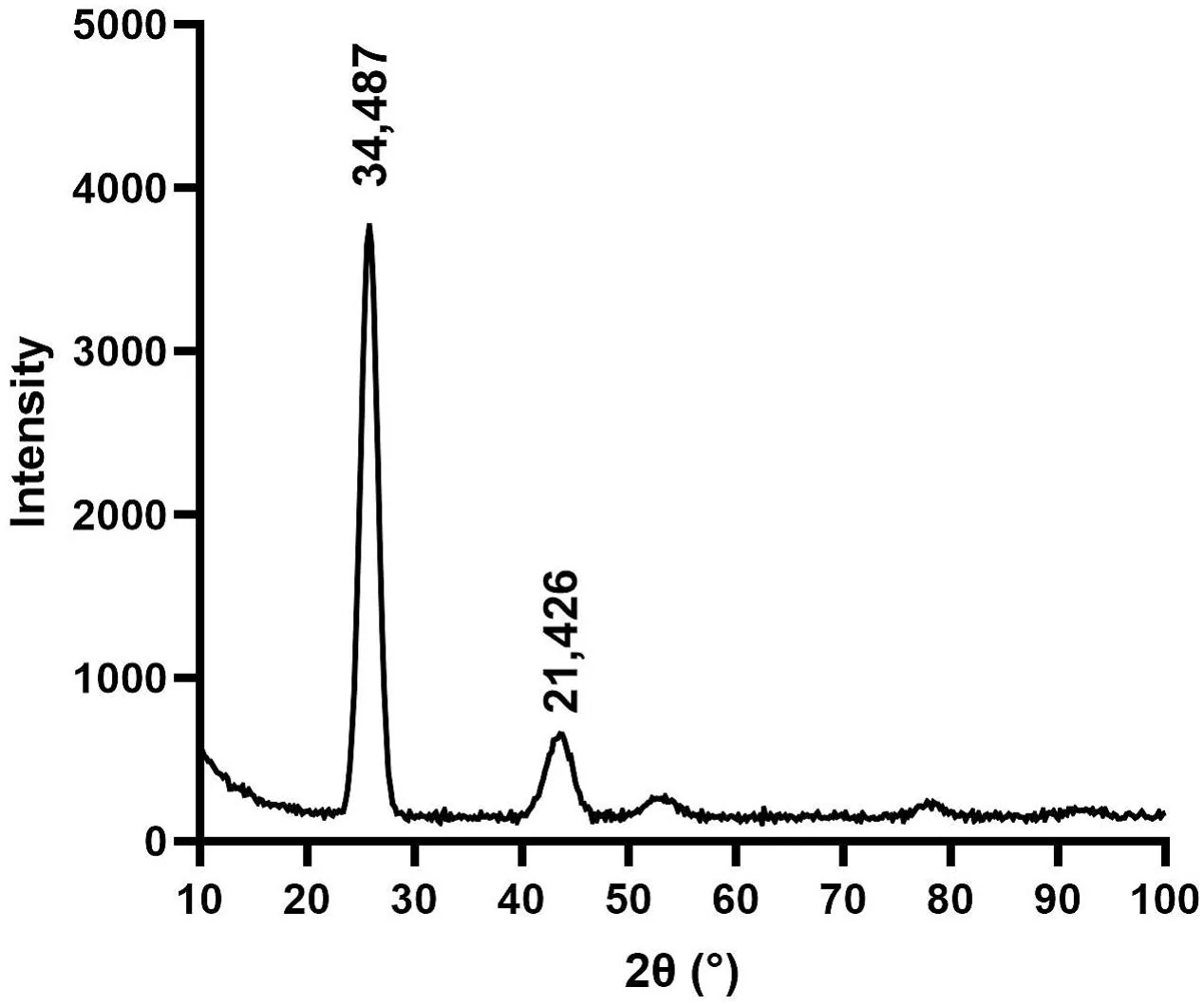
N2 adsorption–desorption isotherms and pore size distribution of MWCNTs indicating mesoporous nature. (1a: Particle size distribution of the synthesized nanoparticles showing a predominant particle diameter of approximately 10–20 nm and a narrow size distribution, indicating good uniformity and limited aggregation.) (1b: XRD spectrum of the synthesized sample exhibiting prominent diffraction peaks at 2θ = 25.49° and 44.21**°**, confirming the crystalline structure and phase purity of the material.).

The XRD pattern of the synthesized material shows a strong diffraction peak at 2 = 26.34 and another minor peak at 2 = 43.16. It demonstrates crystalline nature of the synthesized material. Sharp and intense nature of the diffraction peaks implies that the crystal lattice is well ordered and has high crystallinity. Major crystallographic plane produces the strong peak at 26.34 and another lattice plane gives minor peak at 43.16. High phase purity of the material is evident from the absence of considerable amount of impurity peaks. Subtle wide patterns detected at elevated diffraction angles suggest the existence of a minor amorphous fraction or nanocrystalline domains**. (**Fig. 1**b)** Scanning Electron Microscopy (SEM) analysis further confirmed the multi-walled nature with multi-concentric graphitic layers and hollow tubular architecture. The well-preserved nanotube structure indicated the excellent mechanical stability under the ultrasonic conditions Fig. 2**.** Fig. 2**a** represents the elemental composition of synthesized multiwalled nanotubes.

**Fig. 2:**
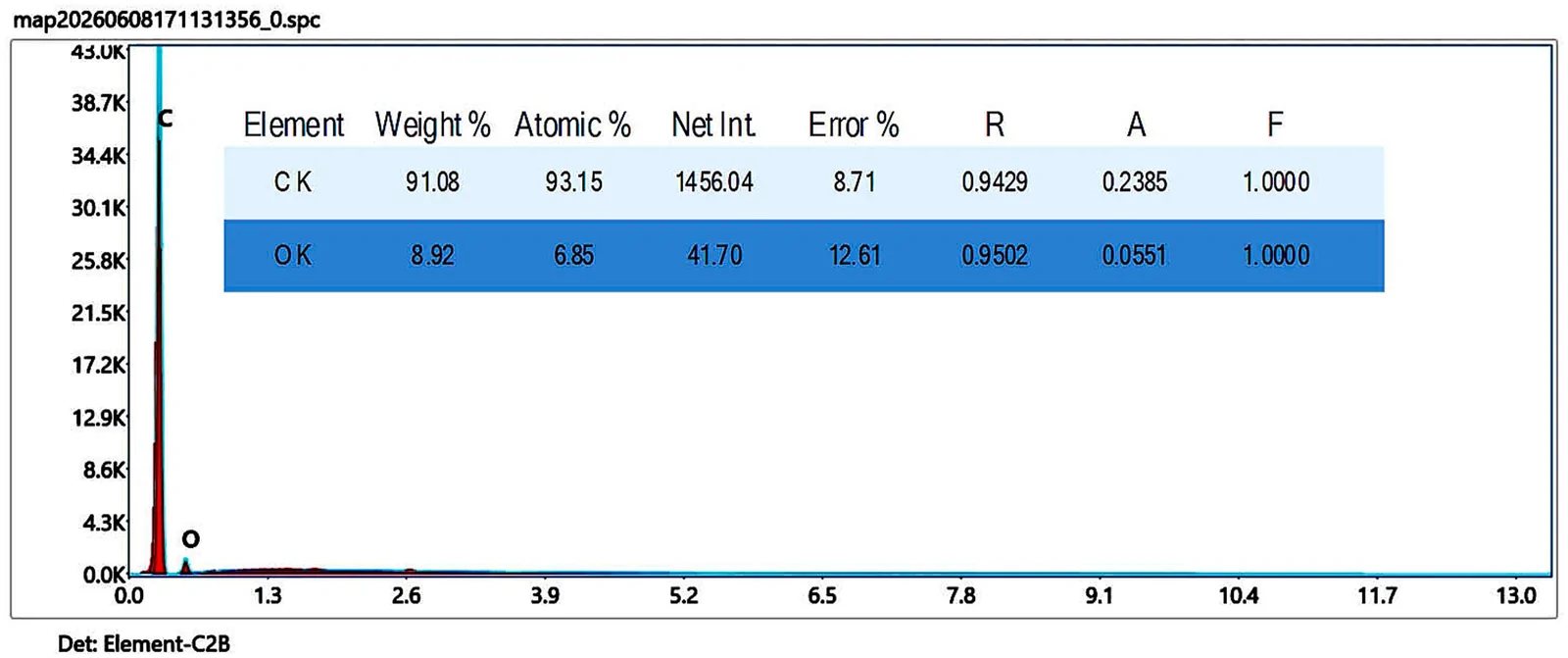

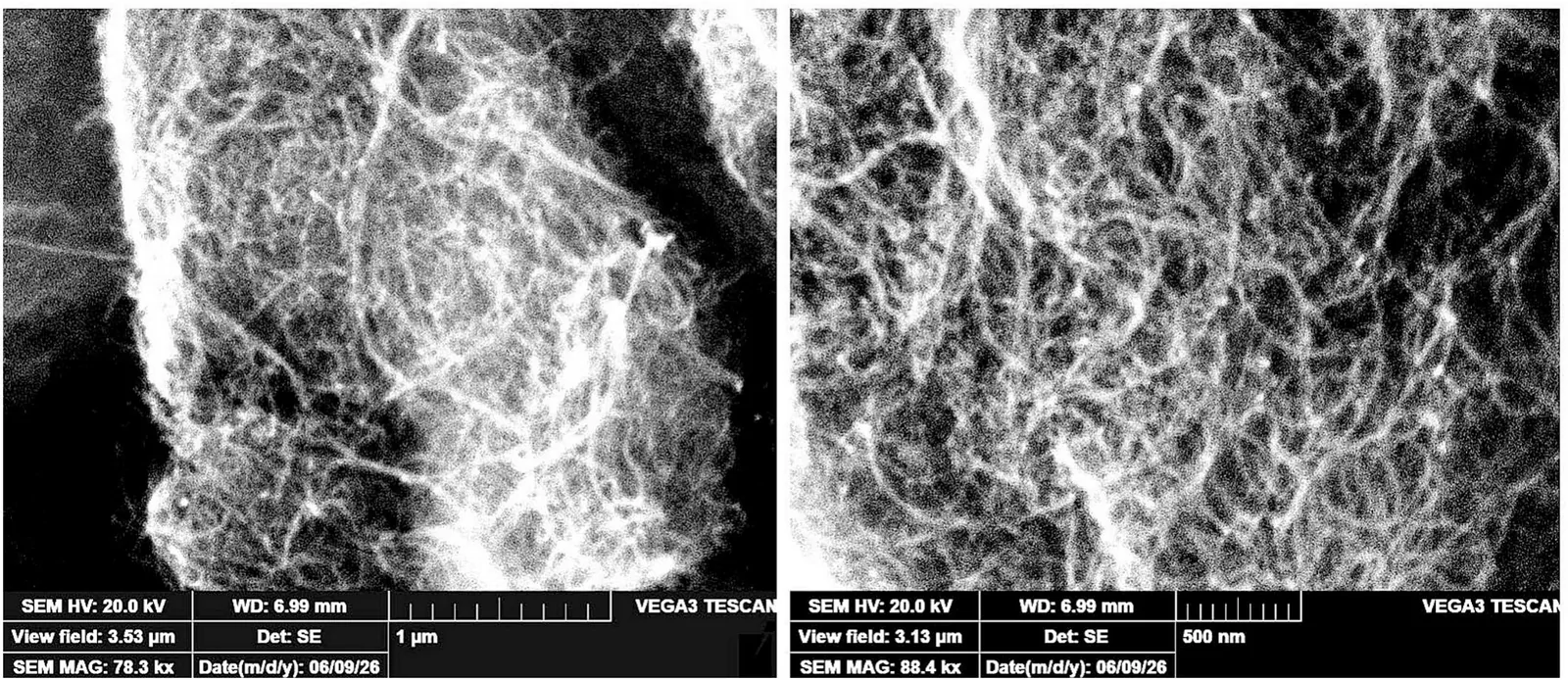
SEM Images and EDX mapping of MWCNTs.

As shown in Fig. 3, the Raman spectrum exhibits distinctive peaks D, G, 2D at 1338 cm^−1,^ 1584 cm ^-1^, 2692 cm ^-1^, respectively. The defectiveness of the graphitic structure and structural disorder are related to the D band. G band represents the in-plane vibrational of the sp 2-hybridized carbon atoms. 2D band shows the graphitic character of the nanotubes. The calculated ID/IG value of 0.82 shows that there is a certain defect density generated through acid functionalization, which benefits sonocatalysis and adsorption [19]. The BET suggests the mesoporous structure has a specific surface area of 210 mg. High surface area can promote effective interaction between the pollutant and ROS because more DES molecules will access the surface (Fig. 4). The pH pzc of MWCNTs value calculated using F-C plot is 5.63, the surface of the catalyst is positive below 5.63 and negative above 5.63. This means the electrostatic interaction between DES molecules and the catalyst surface is more favourable at pH close to neutral condition promoting its adsorption and further sonocatalytic degradation. [12], [26]. The EDS data show the carbon and oxygen was mainly existing, it means that the surface has been effectively oxidized after acid treatment. From FTIR spectra show that hydroxyl, carbonyl and carboxyl functional groups appeared, it enhances its hydrophilicity and catalytic activity.

**Fig. 3:**
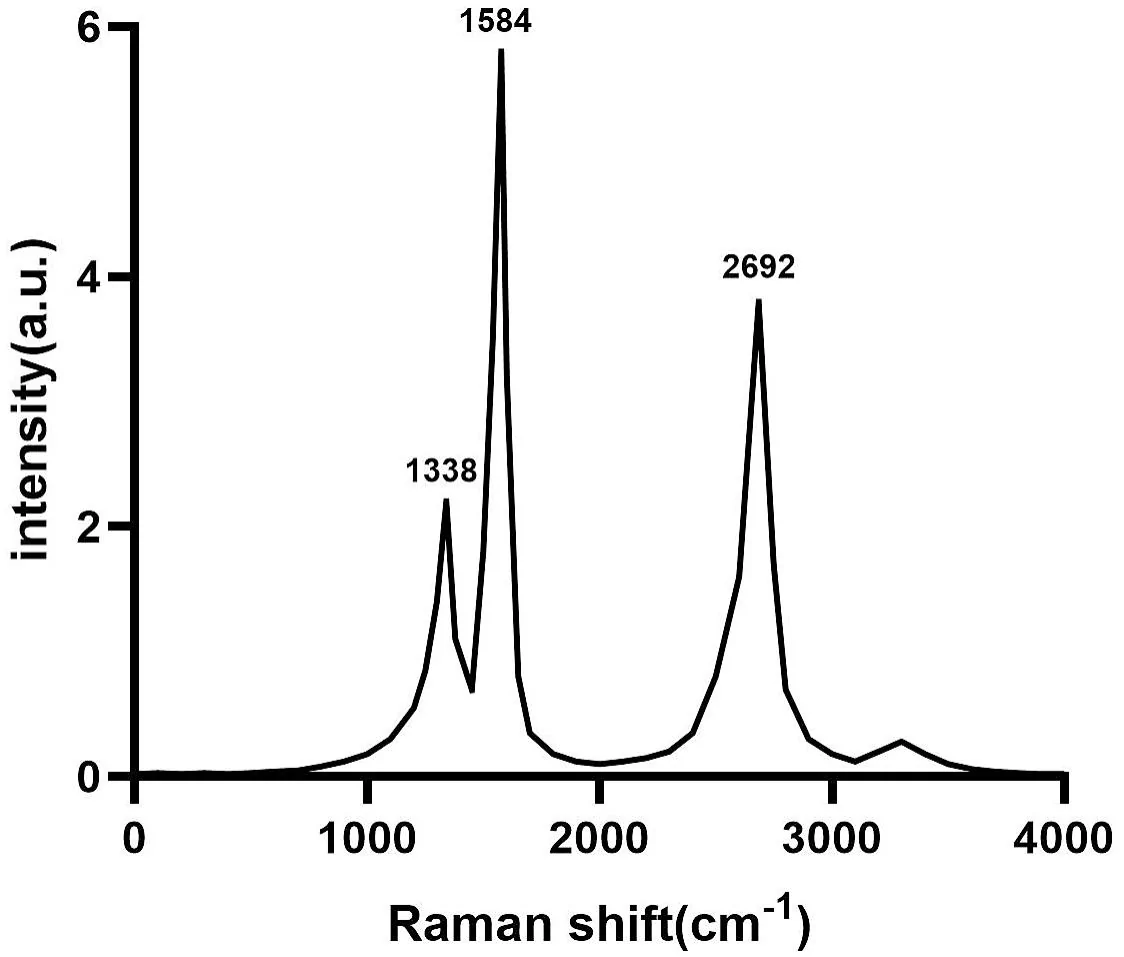
Raman spectrum of the MWCNTs showing the characteristic D band (1338 cm⁻^1^), G band (1584 cm⁻^1^) and 2D band (2692 cm⁻^1^).

**Fig. 4:**
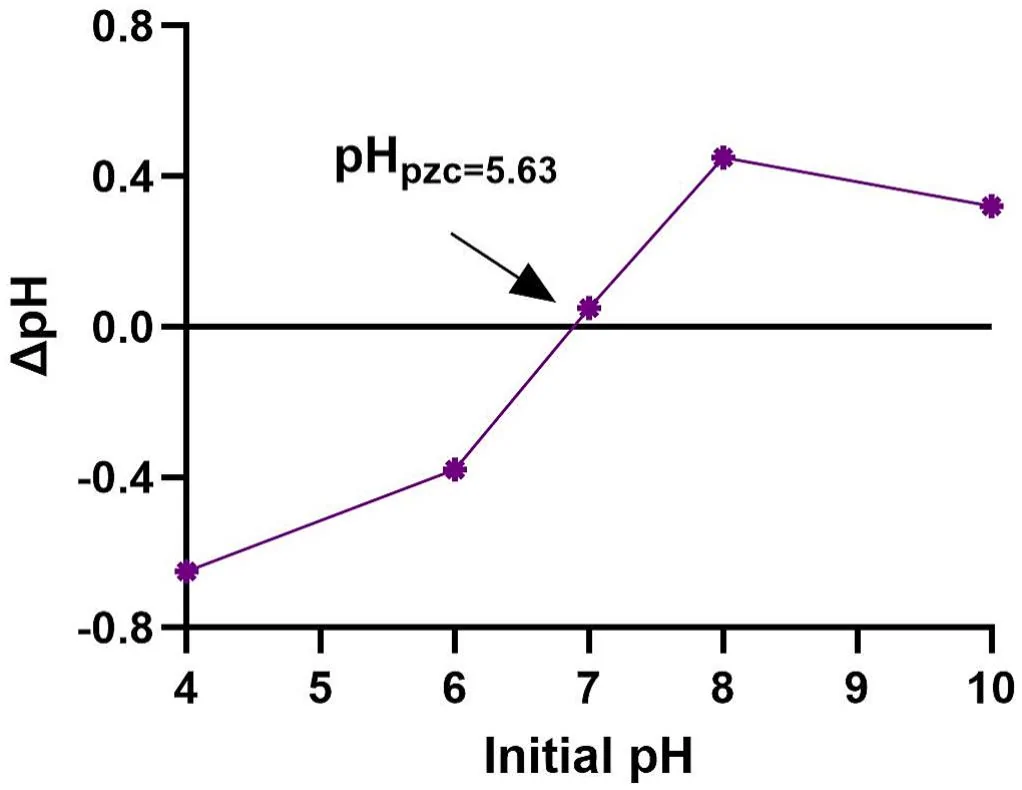
Determination of point of zero charge (pHPZC) of MWCNTs, the pHPZC value is 5.63.

#### 3.1.1 Influence of liquid height on DES degradation

The influence of liquid height on the sonocatalytic degradation of Diethylstilbestrol (DES) in the presence of Multi-Walled Carbon Nanotubes (MWCNTs) was examined at four distinct liquid heights (5, 20, 80, and 240 mm). The degradation of DES was heavily dependent on the liquid height of the reaction media. The influence of liquid height on the sonocatalytic degradation of Diethylstilbestrol (DES) in the presence of Multi-Walled Carbon Nanotubes (MWCNTs) was examined at four distinct liquid heights (5, 20, 80, and 240 mm).

The degradation of DES was heavily dependent on the liquid height of the reaction media. The poor efficiency was greatly improved by lowering the liquid depth. This means better utilization of ultrasonic energy in shallow liquid conditions. The concentration ratio (C/C0) at a liquid height of 5 mm was decreased from 1.0 to about 0.14 after 20 min of treatment, which is equivalent to about 86% degradation. On the other hand, degradation efficiencies of about 76%, 67% and 55% were obtained for liquid heights of 20, 80 and 240 mm respectively. The dependence of the sonochemical efficiency on the liquid height is found to be related to the acoustic attenuation and also spatial distribution of cavitations activity in the reactor. It is the range of the liquid height we chose that allowed finding a condition of obtaining an optimal energy of incident US energy. These results show that a lower liquid depth leads to a more efficient sonocatalytic degradation of DES.

This behaviour can be explained by the attenuation of ultrasonic waves during their propagation through a liquid medium. Lower liquid heights mean more acoustic energy reaches the reaction zone, creating a higher density of cavitation bubbles and a greater production of hydroxyl radicals. On the contrary, the increase of the liquid depth dissipates more energy before the ultrasonic waves reach the catalyst surface, which reduces the cavitation intensity and degradation efficiency. The degradation kinetics corresponded to a pseudo first order model, and the corresponding rate constants are presented in Table 1. The degradation kinetics were fitted to the pseudo-first order model and the calculated apparent rate constants (kobs) along with their R values are shown in Table 1. The excellent R values explain a decent fit of the experimental data on degradation to the pseudo-first order kinetic model.

The maximum apparent rate constant was reached at a liquid height of 5 mm confirming that increased cavitation activity promotes the fast degradation of DES. Similar observations have been reported for sonocatalytic degradation of pharmaceutical contaminants where reduced liquid depth improved acoustic energy transfer and cavitation efficiency.

#### 3.1.2 Influence of stirring speed

The effect of stirring speed on the degradation of DES under MWCNTs ultrasonic irradiation was investigated. The degradation rate showed a great enhancement when the stirring speed was reduced from 0 to 300 rpm. Similar results are observed by [16] shown in (Fig. 5). It means that too vigorous agitation will inhibit the cavitation efficiency. At the low stirring speed, catalyst dispersion was not sufficient which limited the mass transfer of DES molecules, particles, cavitation zone. At the optimal stirring speed of 300 rpm, better mass transfer rate and a homogeneous catalyst suspension allowed the collision of radicals and pollutants. Minor decrease of the degradation efficiency occurred when the stirring speed was 400 rpm. Consequently, the stirring speed selected was therefore convenient in order to detect the point of transition from under suspension and mass transfer to the hydrodynamic over disturbance of the cavitation area; the activity peak at 300 rpm is testimony to the suitable compromise between MWCNT suspension and DES mass transfer to and within cavitation stability.

**Fig. 5:**
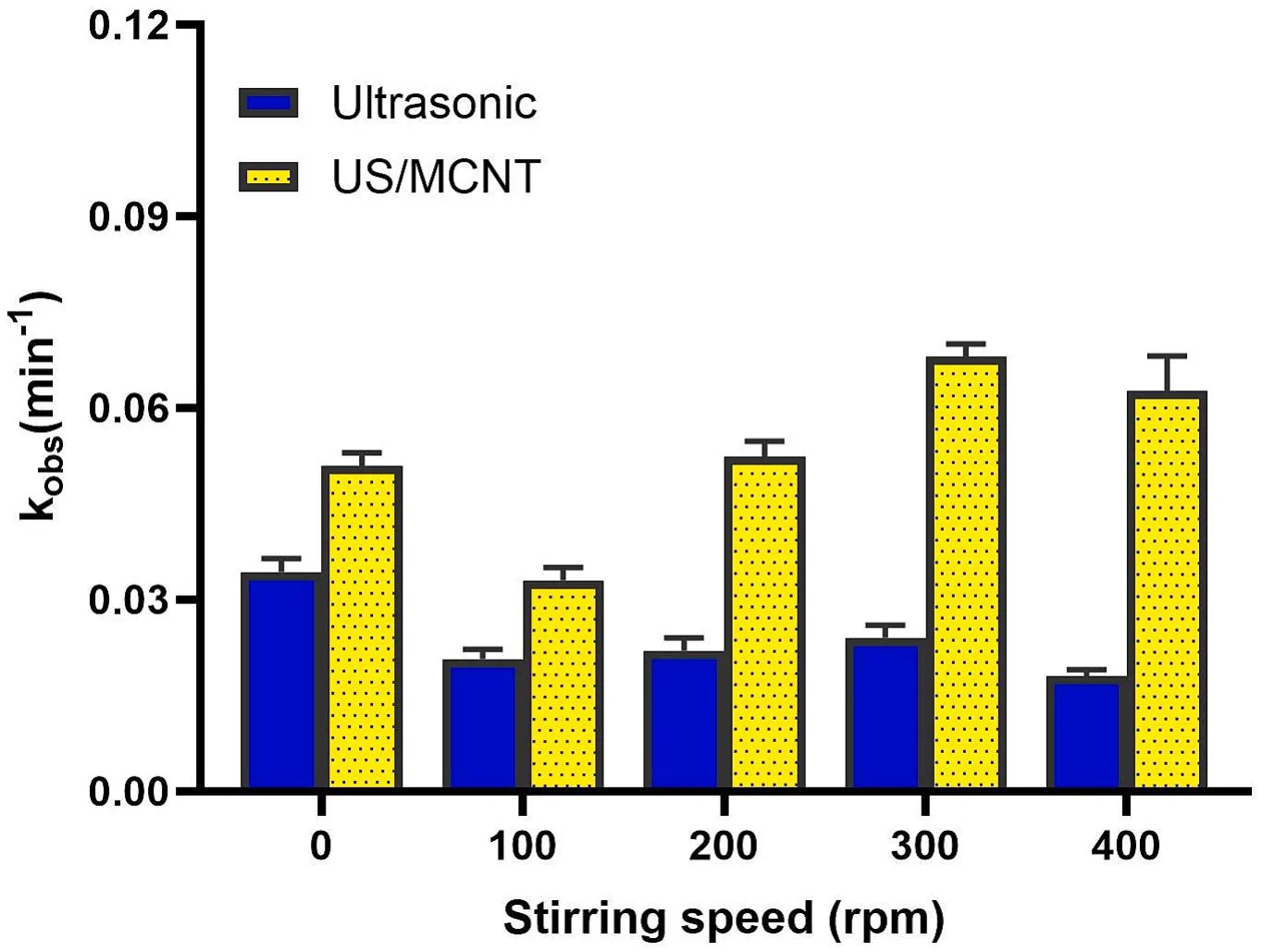
The apparent degradation rate constant (kobs) of DES under ultrasonic irradiation and US-MWCNT system as function of stirring speed SD (n = 3). Statistical significance was evaluated using one way ANOVA (p < 0.05 is statistically significant).

Excess stirring makes cavitation bubbles become unstable and bubble collapsing intensity and generation of hydroxyl radicals decreased. Thus, the optimum hydrodynamic condition to sonocatalytic degradation was at 300 rpm. MWCNT catalyst mass, used for the optimized experimental conditions at the MWCNT loading used ([200 mg/L]), liquid height (5 mm) and speed (300 rpm).

#### 3.1.3 Influence of MWCNT length

The physical dimensions of MWCNTs influence the cavitation behaviour and catalyst dispersion. The nanotubes have been varied in length from short-cut MWCNTs to longer nanotube structures. Degradation performance was higher for shorter MWCNTs because of their improved dispersion and a more available active surface area. Long nanotubes tended to aggregate and entangle, thus decreasing the effective surface area and limiting the interaction with cavitation bubbles. **(**Fig. 6**)** The superior performance of the shorter MWCNTs highlights the significance of catalyst morphology in enhancing the sonocatalytic activity. [12] and [26].

**Fig. 6:**
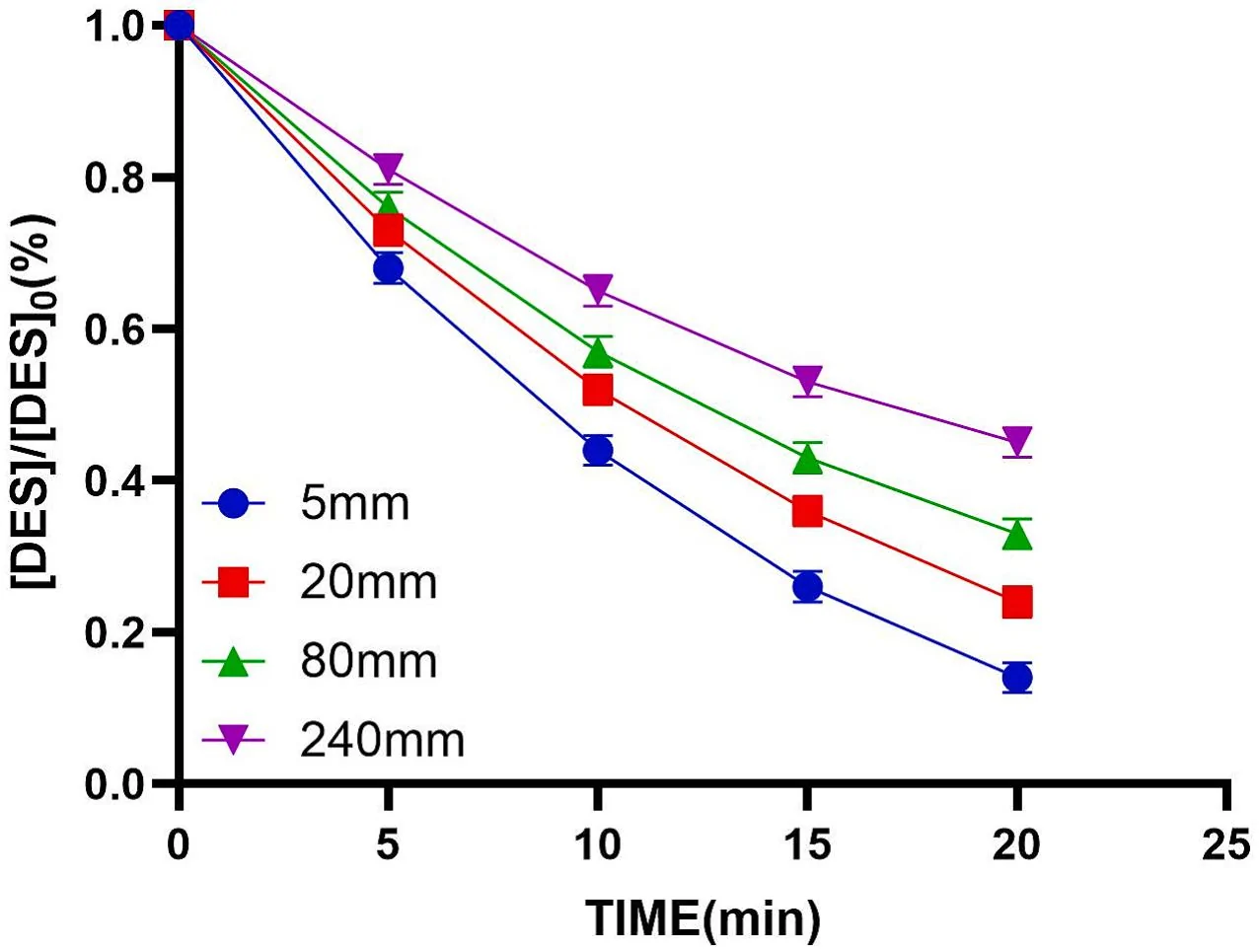
Influence of liquid height on sonocatalytic degradation of DES in US-MWCNT system.

#### 3.1.4 Influence of pH

The pH of a solution affects the surface charge on the catalyst, speciation of pollutant and stability of the radical species. The performance on the degradation of DES was investigated at pH of 4, 7 and 10. The chosen pHs reflect typical acidic, neutral and alkaline conditions in order to study the effects of the interaction of the MWCNT surface charge, DES speciation, hydroxyl-radical availability and the adsorption of pollutants. The trends with regards to the measured pHpzc of 5.63 also suggest that the surface charge of the catalyst must play a significant role in the degradation process. The maximum degradation efficiency occurred at neutral pH (Fig. 7, Fig. 8, Fig. 9). In acid solution, a limited number of hydroxyl radicals are generated and therefore the oxidation reactions could not be promoted as effectively. Under alkaline condition, the generation of hydroxyl radicals is higher but the scavenging effect of hydroxyl radicals and the repulsion effect between catalyst and the pollutant will reduce the overall degradation performance [7], [23]. It might be due to the appropriate ratio of catalyst surface charge, DES adsorption, cavitation efficiency and the stability of hydroxyl radical that makes the best performance achieved under pH 7 and thus presents the excellent properties for the application in practical wastewater treatment.

**Fig. 7:**
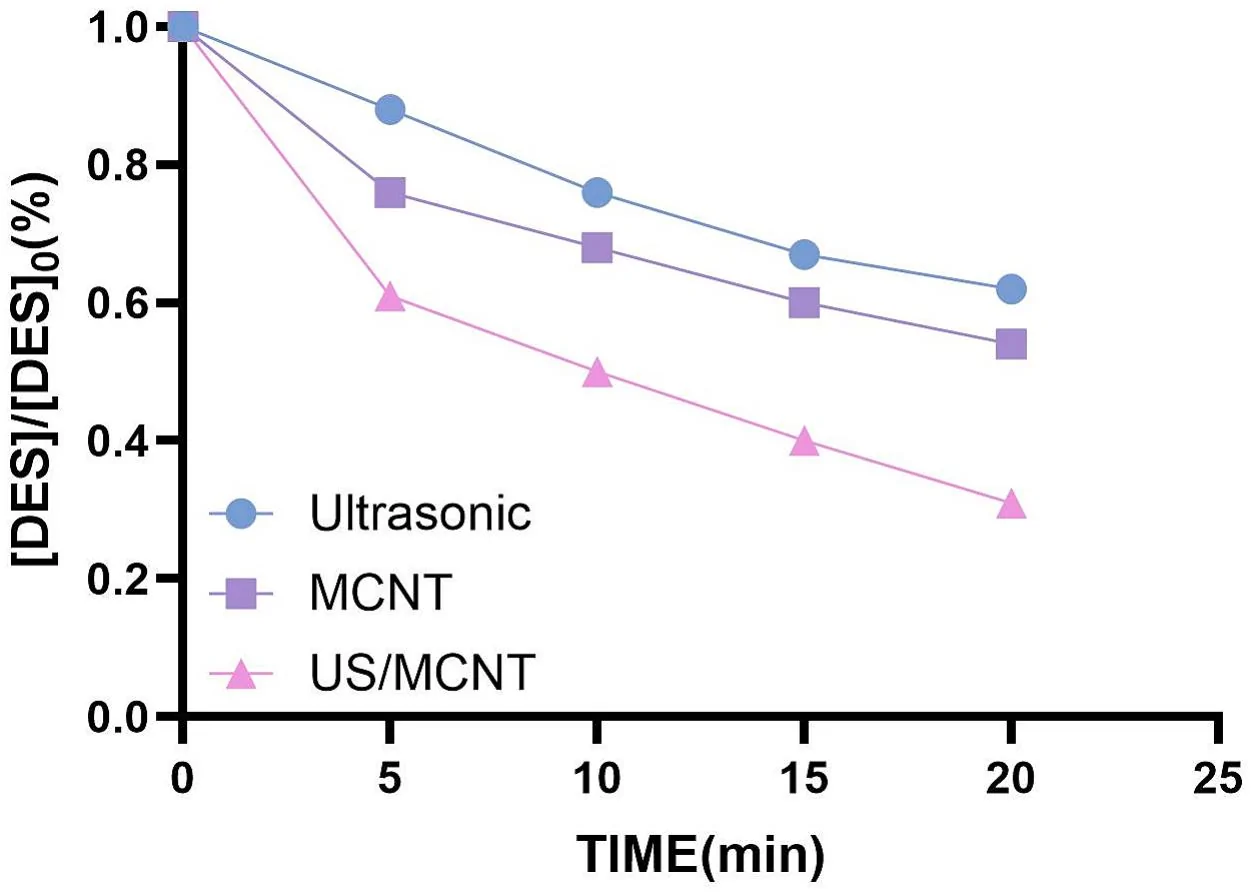
Comparison of DES degradation by ultrasonic irradiation, MWCNTs adsorption and the combined US-MWCNT process.

**Fig. 8:**
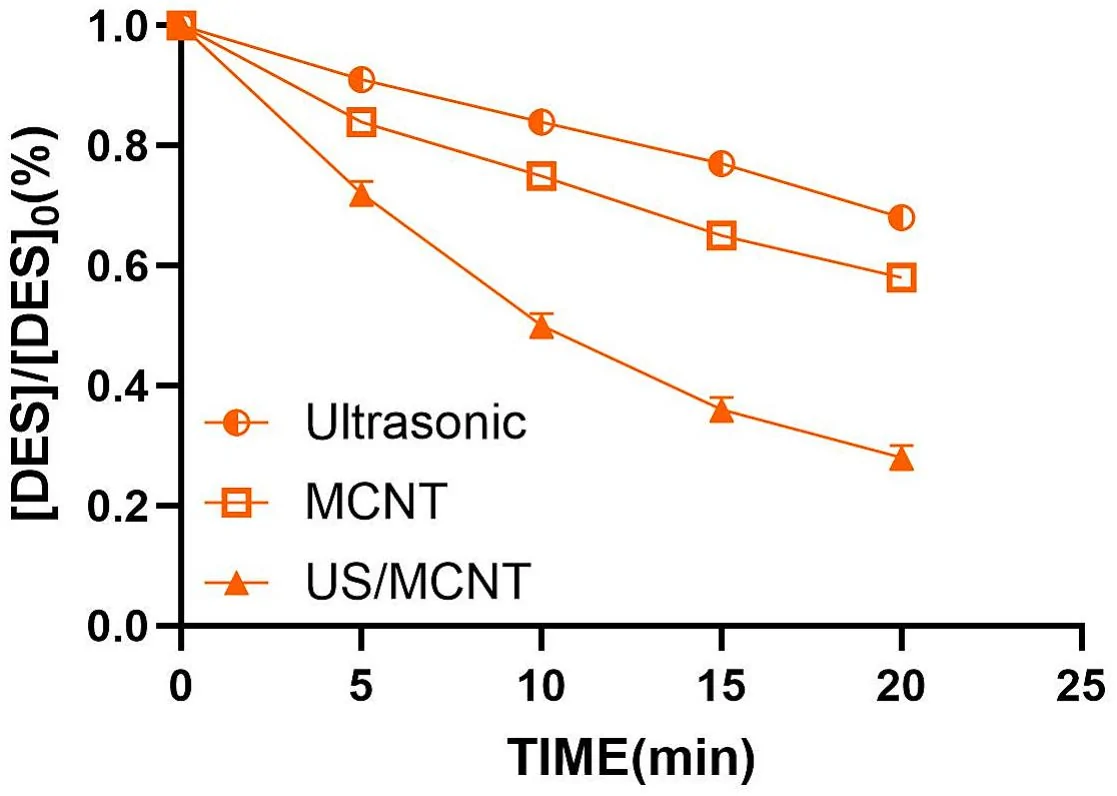
Influence of initial concentration of DES on the degradation efficiency under ultrasonic irradiation, MWCNTs adsorption and US-MWCNT process.

**Fig. 9:**
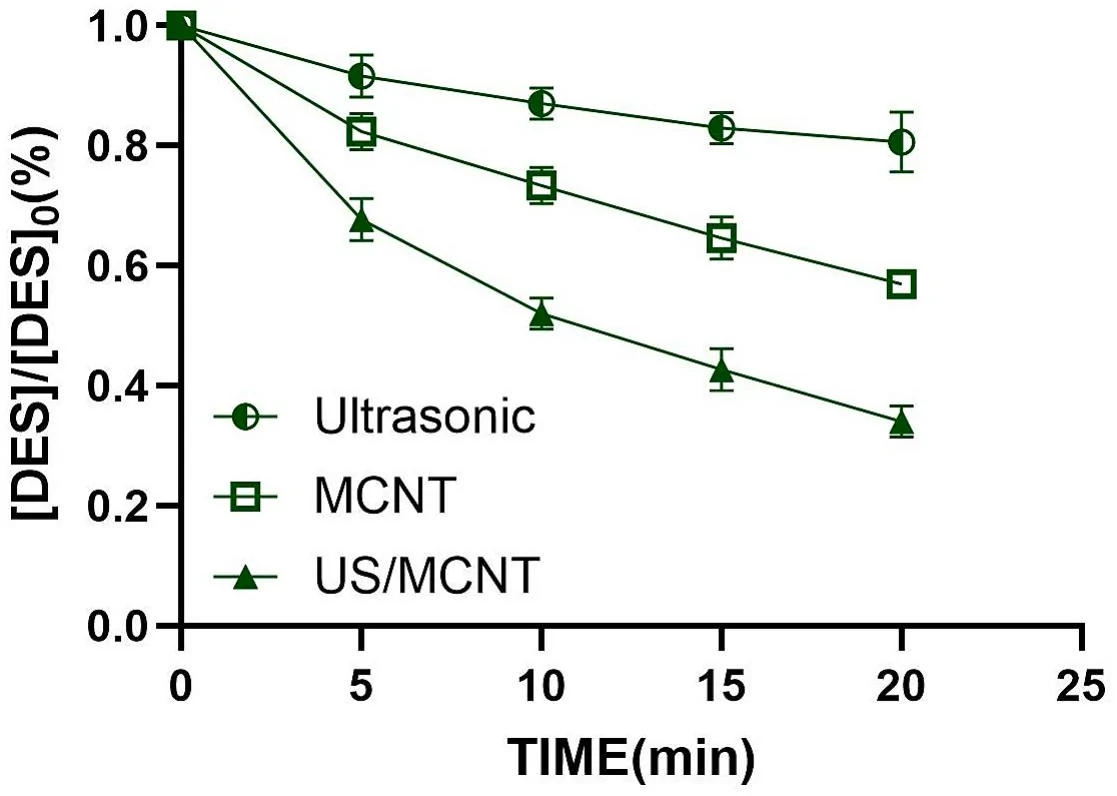
Effect of solution pH on DES degradation by ultrasonic irradiation, MWCNTs adsorption and US-MWCNT process.

#### 3.1.5 The influence of temperature

The influence of reaction temperature on sonocatalytic degradation was examined within the range of 25 to 45°C. It was observed to perform it in an in between temperature range since we are seeking to study the outcome of modest thermal acceleration, and not much vapor pressure amplification that might buffer the collapse stage of cavitation bubbles. With the help of the temperature increase of the apparent rate constant, one can deduct from the resulting dependence. The acceleration of the apparent rate constant is due to many combined factors such as increased frequency of molecular collision and movement, and the kinetics of reaction.

Higher temperature was in favour of the DES degradation efficiency due to the enhanced radical diffusion, increased molecular collision frequency and faster reaction kinetics. The observed pseudo-first order rate constants increased stepwise with increasing temperature. [18] and [23]. However, at temperatures above 45°C the intensity of cavitation may be negatively affected because increased vapor pressure can cushion the collapse of the bubbles. Therefore, moderate temperatures are considered as optimal for an efficient sonocatalytic treatment.

#### 3.1.6 Influence of initial DES concentration

The initial concentration of DES was varied from 2 to 30 μM to study the effect on degradation performance. At lower concentrations DES molecules were readily attacked by hydroxyl radicals, which resulted in rapid degradation kinetics. With the increase of concentration, the degradation efficiency gradually decreased due to the insufficient available radicals with respect to the pollutant load. Nevertheless, the US-MWCNT system always showed a better degradation efficiency than that obtained by the ultrasound alone, indicating a strong synergistic effect between the catalyst and the ultrasonic irradiation. The degradation behaviour was well described by the pseudo-first-order kinetic model at all concentrations. [18] and [22].

#### 3.1.7 Scavenger effect

Radical scavenging experiments were performed in the presence of methanol and *tert*-butanol to investigate which reactive species is responsible for degradation of DES. After addition of scavenger, it was found that degradation efficiency was decrease as compared to without scavenger, which indicates the participation of hydroxyl radical in sonocatalytic process. It was also found that with *tert*-butanol, the degradation of pollutants was more decreased than with methanol, which indicated that bulk phase hydroxyl radical also played a significant role in degradation of pollutants [15], [20], [21]. It is clear from result that oxidation via hydroxyl radical is the major mechanism for the degradation of DES in the US-MWCNT system. **(**Fig. 10, Fig. 11**).**

**Fig. 10:**
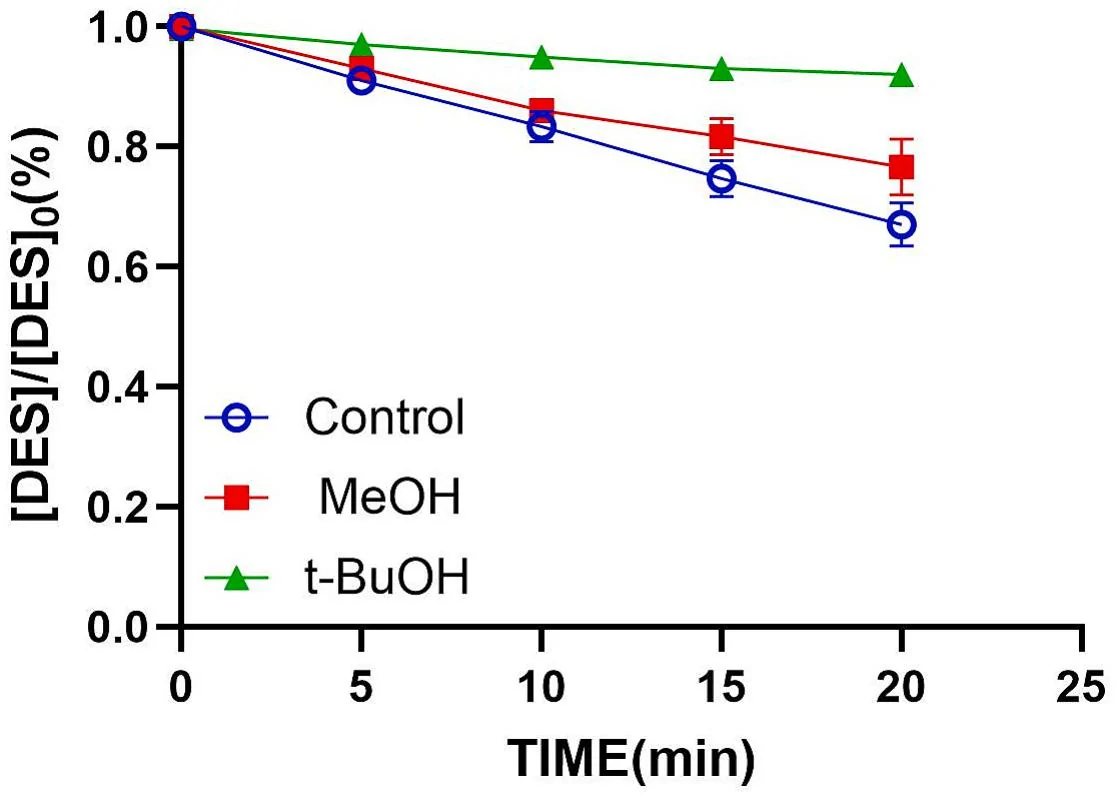
Effect of methanol (MeOH) and *tert*-butanol (t-BuOH) on DES degradation in US/MWCNT system.

**Fig. 11:**
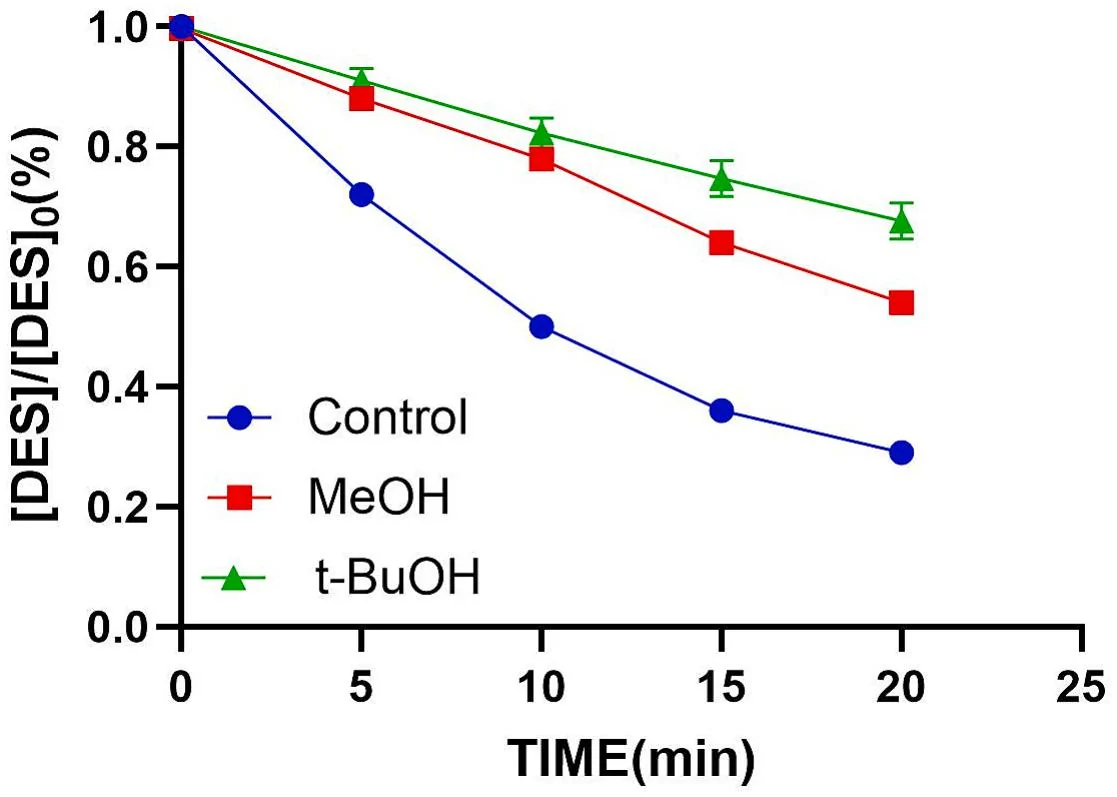
Effect of radical scavengers on the degradation of DES under ultrasonic irradiation only.

#### 3.1.8 Tests for reusability

Long-term stability and practical applicability of MWCNTs were evaluated by five successive degradation cycles [17], [26]. The catalyst maintained above 85% **(**Fig. 12**)** of the initial degradation efficiency for five cycles. The catalyst was very durable under long-term ultrasonic irradiation as the performance was slightly decreased. [17], [12]. The strong graphitic structure of MWCNTs is the main reason for their resistance to ultrasonic erosion and oxidative damage.

**Fig. 12:**
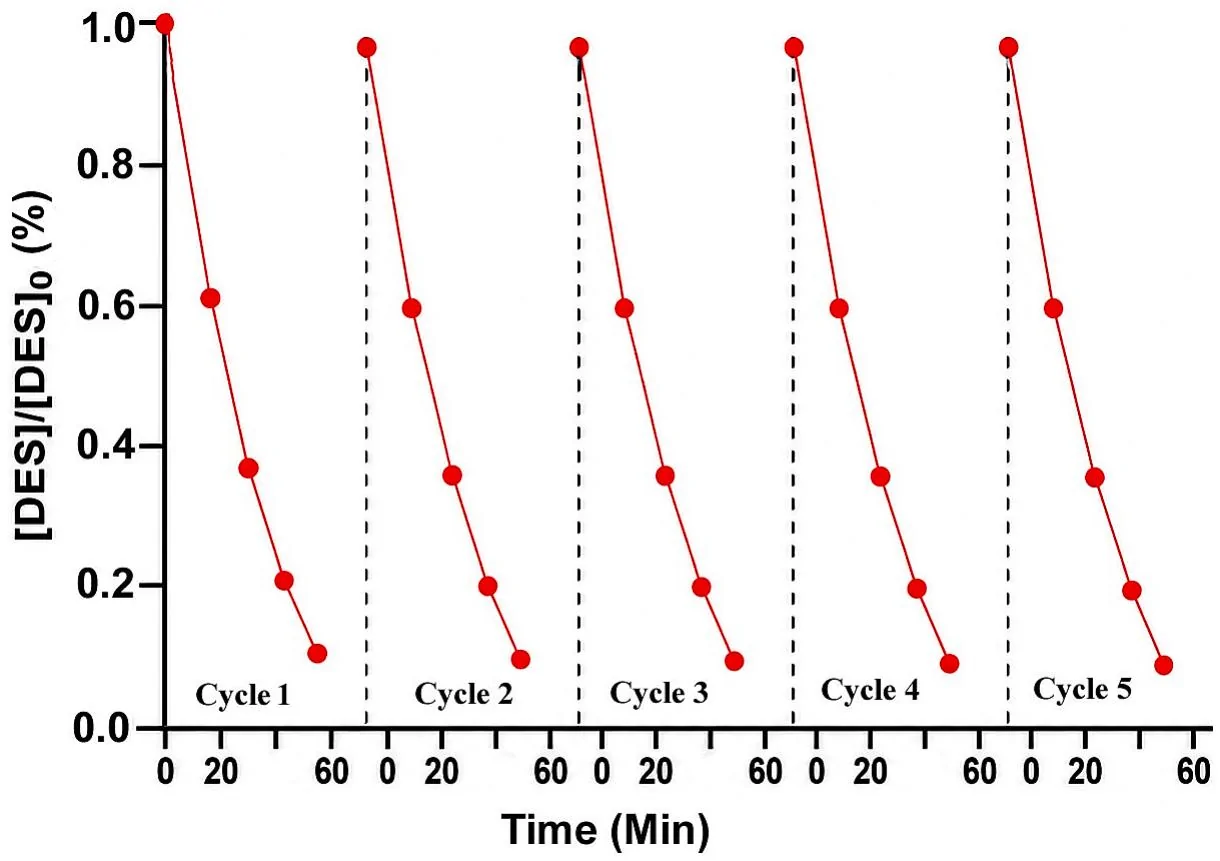
Reusability of MWCNTs for degradation of DES in five successive cycles of sonocatalysis.

#### 3.1.9 Reusability SEM results and EDS mapping for MWCNTs

The structural stability before/after several sonocatalytic cycles was evaluated by FESEM analysis. The pristine MWCNTs exhibited a highly interconnected tubular network. After five cycles, the nanotubes mostly retained their original morphology, but minor fragmentation and surface roughening were observed. No major structural collapse or severe damage was observed. The elements of composition were unchanged when performing a number of consecutive uses as indicated in EDS mapping analysis. The carbon is still the dominant element and the functional groups containing oxygen were kept on the surface of the catalyst. The FESEM and EDS results demonstrate the MWCNTs morphology and the nature of its elemental components remained effectively the same following 5 sonocatalytic processes. We did not conduct Raman and XRD characterization following the 5cycle application, but such post process characterization is recommended and gives conclusive evidence for MWCNT structural persistence. [24].

#### 3.1.10 Treatment of river water

The practical applicability of the sonocatalytic system was evaluated with the river water samples spiked with DES **(**Fig. 13**),** The degradation efficiency was reduced a little compared to the experiments in deionized water, but the US-MWCNT system still removed a substantial amount of DES. The reduction in performance is due to the competition for hydroxyl radicals from the natural organic matter and from dissolved ions [24]. Nevertheless, the system showed high degradation efficiency, which suggests its potential for real-world environmental remediation applications. The low efficiency of degradation in the river water could be explained by the interfering presence of NOM and dissolved inorganic components that act by either outcompeting DES to form • OH radicals, or by affecting pollutant adsorption onto the MWCNT surface. The effect of the presence of each one individually was not examined and further investigation on this topic may be necessary.

**Fig. 13:**
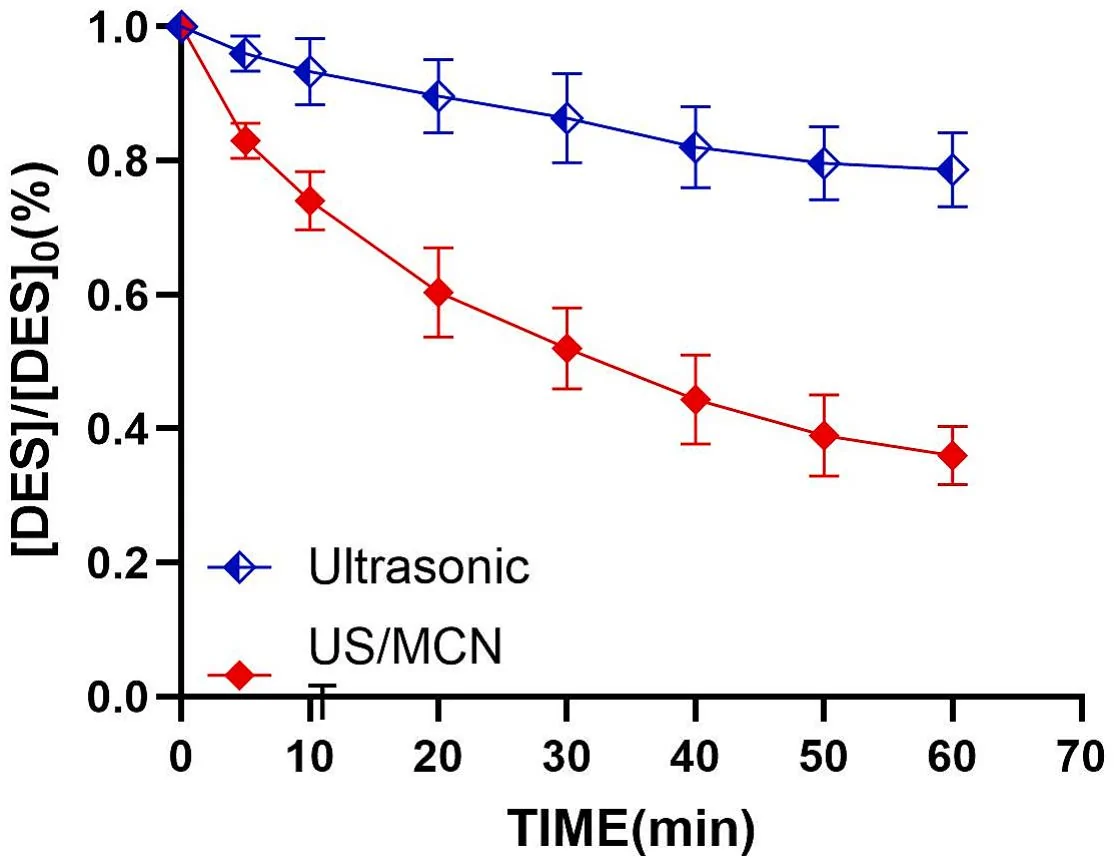
Degradation comparison of DES in ultrapure water and river water by ultrasonic irradiation and US-MWCNT process.

#### 3.1.11 Determination by Calorimetry

To assess actual ultrasonic energy transfer during the treatment, calorimetric analysis was performed. The temperature rise method showed that a significant fraction of the ultrasonic energy was efficiently converted to acoustic cavitation and radical generation. The calorimetric power is in good agreement with theoretical calculation of power required, this indicated that energy within the reactor is not being wasted. Data collected are also useful for industrial production on a larger scale using the sonocatalytic approach. [16], [27].

#### 3.1.12 Comparison with other reported sonocatalytic systems

The performance of the US-MWCNTs system was also compared with the recently reported sonocatalytic approaches for the removal of endocrine-disrupting and other persistent organic contaminants. The presence of MWCNTs in conventional ultrasonic treatment alone provides additional sites for pollutant adsorption and also aids in the interaction between DES molecules and the ultrasound generated reactive species. The improved degradation in the present study with the US-MWCNTs system shows the advantage of combining the acoustic cavitation with a carbon-based nano catalyst. The synergistic effect between ultrasound and MWCNTs is in agreement with the growing use of carbonaceous materials in sonocatalytic advanced oxidation processes. However, direct comparison among reported sonocatalytic systems should be done with caution since the degradation performance is strongly affected by ultrasonic frequency and power, reactor configuration, pollutant concentration, catalyst dosage, solution volume, reaction time and water-matrix composition. Thus, the present MWCNT-assisted system offers a promising alternative for the sonocatalytic treatment of DES and related endocrine disrupting contaminants, but further optimization of the reactor design and energy utilization will be needed for practical application.

#### 3.1.13 Key degradation process

Hydroxyl radical generation efficiency was enhanced when MWCNTs were present. This finding was supported by EPR and benzoic acid probe experiments [21], [25]. Ultrasound irradiation creates an acoustic cavitation which produces hotspots that promote water dissociation as the.

H_2_O → •OH + •H **(**Fig. 14**).**

**Fig. 14:**
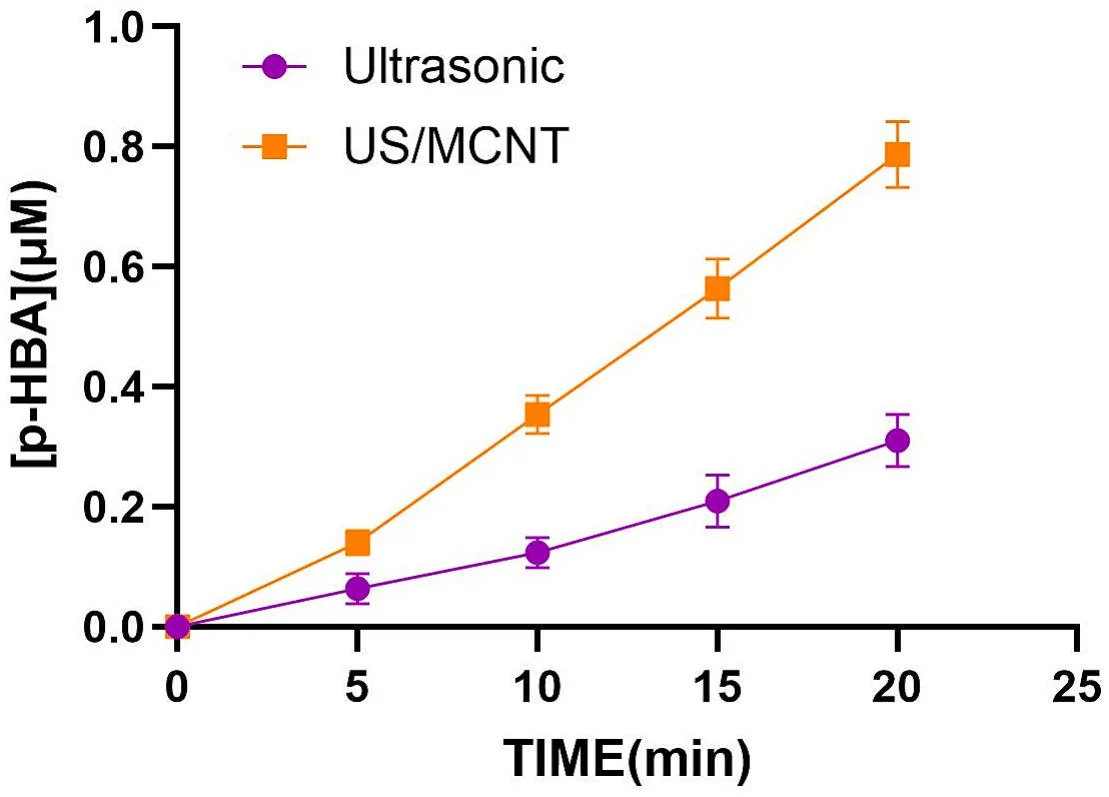
Time-dependent formation of p-hydroxybenzoic acid (p-HBA) during ultrasonic and US/MCNT-assisted degradation processes.

MWCNTs are cavitation nuclei and facilitate bubble inception and collapse. At the same time, DES molecules are adsorbed on the catalyst surface by means of π–π interactions, which brings them closer to reactive radicals. [22], [28]. The produced hydroxyl radicals are able to attack the aromatic rings and phenolic groups of DES and hydroxylation, ring opening, fragmentation and subsequent mineralization take place. Finally, sequential oxidation reactions convert DES into low-molecular-weight organic acids, CO2 and water. Synergistic effect of Ultrasound and MWCNTs improves the cavitation intensity, radical production, pollutant adsorption and oxidation efficiency. MWCNTs also function as heterogeneous cavitation nuclei owing to the presence of active sites on the MWCNTs surfaces for bubble nucleation. Cavitation-bubble collapse and nucleation processes can be assisted during sonication owing to the MWCNTs, leading to a localized ultrasonic chemical reaction which increases hotspots to facilitate water splitting and form hydroxylation radical (OH). The pollutant molecules adsorbing on the MWCNTs surface are brought nearer to the reactive species and result in increasing the interaction between them. From a practical standpoint, the US-MWCNTs system offers the potential for economic advantages through catalyst reuse and simplicity of operation. However, the energy consumption in the ultrasonic treatment is an important factor in the overall cost of the treatment. Therefore, detailed techno-economic analysis and assessment of energy-efficiency are required before large-scale application.

## 4 Mechanism of ultrasound-assisted degradation of Diethylstilbestrol (DES) using multi-walled carbon nanotubes (MWCNTs)

The destruction of DES under the conditions of MWCNTs under ultrasonic irradiation is primarily associated with the combined effects of ultrasonic cavitation, radical oxidation and sonolytic degradation. With the propagating ultrasound waves in the aqueous medium, numerous small cavitation bubbles are created and collapsed which leads to the creation of the extreme localized hot spots (as high as around 5000 K) and large pressure (over 1000 atm) where extremely high energetic microenvironment existed in the aqueous solution, thereby promoting the sonolysis of the water molecules to produce highly reactive oxygen species (ROS) including hydroxyl radical (OH) and hydrogen radical (H), both of which contribute significantly in the destruction of the pollutants.

Being one of the most strongly oxidizing agents, the generated hydroxyl radical efficiently attacks the DES molecules in the aqueous solution. Due to the unique structure of MWCNTs, their large specific surface area for bubble formation and support is enabling them perfect cavitation nucleus. Moreover, the adsorption of the DES molecules onto MWCNT surface plays an effective role in increasing the proximity of pollutes to react species and thereby enhancing the collisions between pollutants and generated radicals. Besides that, MWCNTs which has extensively delocalized electronic system, can readily promote electron transfer process, which then further stimulates the generation of the radicals, thus increasing the oxidation efficiency of the system.

Under the oxidation by the hydroxyl radical on the MWCNT surface, DES molecules are destroyed, which in fact means the reaction involving breaking the carbon–carbon bonds, the hydroxylation of the ring and ethylenic bridge. The aromatic structure becomes more and more instable, which can be oxidized to some oxygen-containing organic intermediates. Meanwhile, thermal impacts and mechanical forces due to the collapse of cavitation bubbles can be direct participants in promoting sonolytic degradation of DES. The destruction of DES with both radical oxidation and sonolysis leads to the fragment of the parent organic matter into small organic molecules.

Throughout the entire degradation process, the degradation products (intermediate organic matter) generated continue to undergo oxidation by the hydroxyl radical and other species generated under ultrasonic irradiation. The reaction proceeds in a sequence of steps and ultimately breaks the organic framework to relatively low molecular weight organic molecules, aldehydes, ketones and others. Continuous oxidation can mineralize these organic matters into eco-friendly matters such as CO, HO and inorganic ions. Due to the constant production of active radicals throughout the sonolysis process, the continuous degradation and mineralization can be performed. The reaction path for Des degradation in the US-MWCNTs system is hypothesized through multiple steps of oxidative transformation; the first step of the mechanism is assumed to be attacking mainly by hydroxyl radical. These first transformation reaction would likely occur on the phenolic and aromatic ring structure of Des through hydroxylation and oxidation, subsequently a fragmentation and split of Des molecule with a result of low molecular oxygenated intermediate is likely formed followed by subsequent oxidation for lower molecular weight product formed into organic acid. Organic acid can further be oxidized further toward mineralization product (CO and HO and ions), since the first transformations are all involving with hydroxyl radical attack on DES and degradation is continuously occurred, it assumed that above transformation is correct.

But still, individual transformation product could not be detected by LC-MS/GC–MS in current analysis, individual transformation product is only a hypothetical compound, not found in experiments. Hence, we cannot conclude the environmentally benign of the treated solution based on DES remove only. Future study about identification of every transformation product and toxicity analysis on all products formed is need to find the truth. The improved degradation efficiency in the ultrasound-MWCNT system is found to be caused by the synergistic effect of sonocavitation, active species generation, accumulation of the pollutants onto MWCNT surface and accelerating oxidation reaction by MWCNTs. It shows a significantly high efficiency than pure sonolysis process, revealing a practical potential using MWCNTs mediated ultrasonic process to treat the EDCCs in aquatic environment. The proposed degradation mechanism for DES in the ultrasound-MWCNT system is presented in the (Scheme 1).

**Scheme 1:**
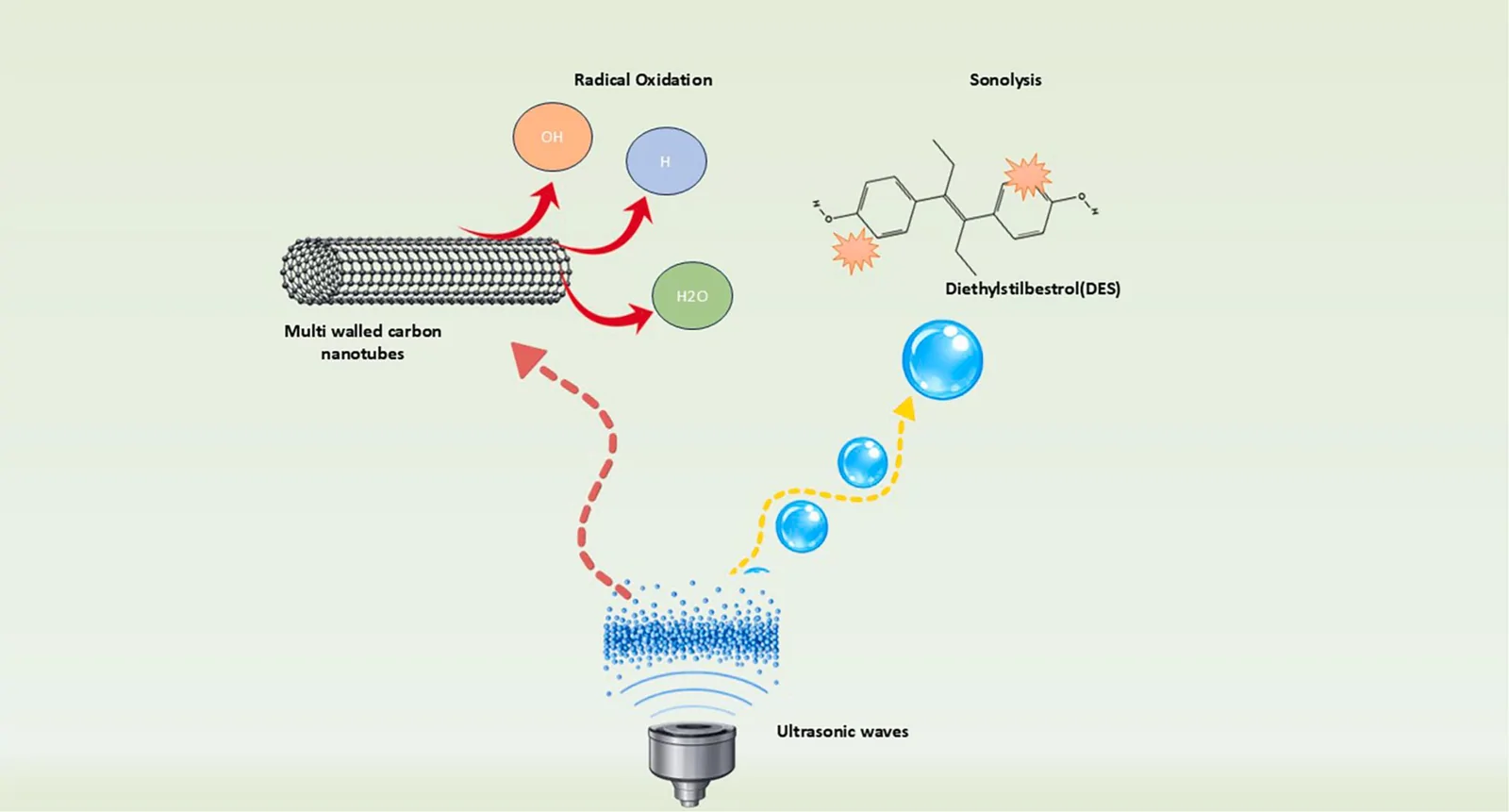
Proposed mechanism of ultrasound assisted degradation of DES with MWCNTs acoustic cavitation, radical generation, adsorption and oxidative degradation of DES.

## 5 Conclusion

In the present study, a convenient MWCNT-supported sonocatalytic system was successfully prepared for the decomposition of diethylstilbestrol (DES), an obstinate endocrine-disrupting compound, often detected in aqueous environment. The integration of ultrasonic irradiation with MWCNT enhanced much higher degradation activity when compared with alone ultrasonic irradiation or only catalyst adsorption, which proved that integrated sonocatalysis has significant effective activity. The appropriate physicochemical properties of MWCNT were thoroughly demonstrated by various characterization analyses. The mesoporous structure with most pore sizes located in 35–45 nm was confirmed by BET measurement and D, G and 2D peaks are at 1338, 1584 and 2692 cm–1 respectively in Raman spectroscopy. The calculated ID/IG ratio of 0.82 indicated the presence of defect sites that contributed to enhanced adsorption and catalytic activity. Furthermore, the pHPZC value of 5.63 suggested favourable surface interactions between DES molecules and the catalyst under near-neutral conditions.

The operational parameters including liquid height, stirring speed, pH, temperature and initial pollutant content had a significant effect on the degradation efficiency of DES.The best degradation performance (5 mm liquid height and 300 rpm stirring speed) was obtained under the tested conditions, suggesting the significance of ultrasonic energy transfer and catalyst distribution.The enhanced degradation of US/MWCNT system was attributed to synergistic effects of acoustic cavitation, improved adsorption of DES onto catalyst surface and continuous generation of reactive oxygen species.Radical scavenging tests indicated that hydroxyl radicals (•OH) were the primary oxidative species responsible for the degradation of DES.The presence of degradation intermediates suggested that the degradation route involved hydroxylation, aromatic ring oxidation, fragmentation and subsequent mineralization events.

The efficacy of the devised system was confirmed using river water samples contaminated with DES. The presence of dissolved ions and natural organic matter caused a small decrease in the degradation efficiency, but still a large removal of pollutants was achieved, demonstrating the robustness of the process under realistic environmental conditions. Moreover, the catalyst showed good stability and reusability, maintaining more than 85% of its initial activity after five consecutive treatment cycles. The FESEM and EDS studies confirmed that the nanotube shape and composition was maintained over a number of uses. The results suggest that MWCNT assisted sonocatalysis is a feasible, ecofriendly and highly effective advanced oxidation technique for the elimination of endocrine disrupting pollutants from aquatic environments and holds great potential for future wastewater treatment application. Degradation performance was promising, however, there are still challenges regarding ultrasonic energy consumption, scale-up of the reactor and optimization of acoustic energy distribution in larger treatment volumes. Further studies are needed to evaluate the energy efficiency, long-term stability of the catalyst and the performance under different real wastewater conditions before practical large-scale application.

## CRediT authorship contribution statement

**Manman Sun:** Writing – review & editing, Conceptualization. **Yihan Sun:** Writing – review & editing, Data curation. **Jingzhen Wang:** Writing – review & editing, Investigation. **Shengkun Tang:** Writing – review & editing, Formal analysis. **Hongyu Chen:** Writing – original draft, Project administration.

## Funding

This work was supported by Doctoral Scientific Research Foundation of Zhoukou Normal University (ZKNUC2022012), Key Research Projects of Henan Higher Education Institutions (22A150057), College Student Innovation and Entrepreneurship Training Program of Zhoukou Normal University (S202510478035).

## Declaration of competing interest

The authors declare that they have no known competing financial interests or personal relationships that could have appeared to influence the work reported in this paper.
